# Supplementary material for: A high-resolution mRNA expression time course of embryonic development in zebrafish
Source: eLife. 2017 Nov 16;6:e30860. doi: 10.7554/eLife.30860 (PMC5690287; doi:10.7554/eLife.30860)
Supplement: Supplementary file 6. [file elife-30860-supp6.zip › biolayout-clusters-files/Cluster098-genes.html]

Cluster098


# Cluster098: Genes

| Ensembl ID | Gene Name | Chr | Start | End | Biotype |
| --- | --- | --- | --- | --- | --- |
| ENSDARG00000077791 | BRSK2 (1 of many).1 | 7 | 49051940 | 49322247 | protein\_coding |
| ENSDARG00000016687 | acadvl | 7 | 20046365 | 20081548 | protein\_coding |
| ENSDARG00000012914 | arhgef4 | 2 | 16530145 | 16690452 | protein\_coding |
| ENSDARG00000045747 | cped1 | 4 | 10617858 | 10705736 | protein\_coding |
| ENSDARG00000068208 | def8 | 18 | 31302658 | 31481351 | protein\_coding |
| ENSDARG00000018397 | hpca | 19 | 33036110 | 33123980 | protein\_coding |
| ENSDARG00000008639 | kifap3a | 20 | 34517744 | 34550744 | protein\_coding |
| ENSDARG00000037324 | klhl17 | 23 | 23255486 | 23288048 | protein\_coding |
| ENSDARG00000034871 | mid2 | 14 | 27936333 | 28084563 | protein\_coding |
| ENSDARG00000015891 | ptprea | 12 | 43313076 | 43413619 | protein\_coding |
| ENSDARG00000002840 | si:dkey-28b4.8 | 1 | 43950424 | 44002958 | protein\_coding |
| ENSDARG00000076393 | tmem65 | 19 | 177065 | 187781 | protein\_coding |
| ENSDARG00000056481 | vat1 | 3 | 19764592 | 19822034 | protein\_coding |
